# A Preliminary Study of the Relationship between Perceived Racism and Cardiovascular Reactivity and Recovery in Native Hawaiians

**DOI:** 10.1007/s40615-018-0463-4

**Published:** 2018-03-06

**Authors:** Andrea Hepuapo‘okela Hermosura, Stephen N. Haynes, Joseph Keawe‘aimoku Kaholokula

**Affiliations:** 1 Department of Native Hawaiian Health, John A. Burns School of Medicine, University of Hawai‘i at Mānoa, 677 Ala Moana Boulevard, Ste 1016, Honolulu, HI 96813 USA; 2 Department of Psychology, University of Hawai‘i at Mānoa, 2530 Dole Street, Sakamaki C 400, Honolulu, HI 96822 USA

**Keywords:** Native Hawaiians, Perceived racism, Reactivity, Recovery, Cardiovascular disease, Stress

## Abstract

Native Hawaiians, compared to other ethnic groups in Hawai‘i, have significantly higher mortality rates and die at a younger average age from cardiovascular disease (CVD). This may be partially explained by elevated cardiovascular responses to racial stressors. Our study examined the degree to which blood pressure (BP) and heart rate (HR) reactivity and recovery, and ratings of subjective distress to racial stressors, differ as a function of Native Hawaiian college students’ levels of perceived racism. This study had three phases. Phase 1 involved the development of a blatant and subtle racial stressor. Phase 2 involved assigning 132 students into high- or low-perceived racism groups based on scores on two perceived interpersonal racism measures. Phase 3 involved a psychophysiology laboratory experiment conducted with 35 of the 132 students. BP, HR, and subjective distress were measured during exposure to the blatant and subtle racial stressors. Systolic blood pressure (SBP) recovery following exposure to both stressors was significant for both groups. Although not significant, three trends were observed among the high-perceived racism group, which included: (1) greater reactivity to exposure to the subtle stressor than to the blatant stressor, (2) incomplete HR recovery following exposure to both stressors, and (3) incomplete SBP and diastolic blood pressure recovery following exposure to the subtle stressor. Participants also reported significantly greater subjective distress following exposure to the blatant than to the subtle stressor. Specific interventions, such as increased self-awareness of physiological responses to racial stressors, targeted at at-risk individuals are necessary to reduce a person’s risk for CVD.

## Introduction

Perceived racism negatively impacts the health and well-being of many ethnic and racial minority groups in the USA, who often experience it daily [1] and in various settings (e.g., work, school, restaurants) [2]. Previous research suggests that higher levels of perceived racism are associated with reduced mental and physical health status [1, 3–16]. This may be due to its relationship with other social determinants of health, such as limited access to economic resources and quality health care [17]. Racism has been defined differently in the literature. It is sometimes used interchangeably with racial discrimination, race and discrimination, oppression, and perceived discrimination [18]. For the purposes of this study, *racism* was operationally defined as “the beliefs, attitudes, institutional arrangements, and acts that tend to denigrate individuals or groups because of phenotypic characteristics or group affiliation” [19]. Therefore, *perceived racism* is defined as the “subjective experience of thoughts, beliefs, and actions by individuals or institutions that lead to or result in the denigration of an individual or group because of ethnic group affiliation or phenotypic characteristics” [20].

Native Hawaiians have a long history of experiences with racial discrimination and negative health outcomes. Since the arrival of Westerners to Hawai‘i in 1778, Native Hawaiians experienced drastic depopulation due to infectious diseases and changes to their worldview, economic status, physical health, cultural practices, and political structure [21]. Currently, Native Hawaiians are among the most socially and economically disadvantaged ethnic groups in Hawaiʻi. They are also more likely than other ethnic groups to be exposed to a greater number of environmental stressors (e.g., poor housing, low-paying jobs), experience higher psychological distress (e.g., depression, sense of helplessness), and encounter more acculturative stressors and racial discrimination [21–23].

### Cardiovascular Disease among Native Hawaiians

Not only is the negative impact of racism indicated by the social and economic statuses of Native Hawaiians, but racism could also have a significant negative effect on their physical health. In Hawaiʻi, cardiovascular disease (CVD)-related mortality rates are disproportionately higher for Native Hawaiians than for other ethnic groups [24]. The only exception is Filipinos who also experience similar discrimination [24]. The mortality rates are estimated to be 313.1 per 100,000 for Native Hawaiians compared to 205.3 per 100,000 for the general population of Hawai‘i [24]. Native Hawaiians die an average of a decade younger from CVD compared to the other major ethnic groups in Hawai‘i [24]. They also have higher rates of CVD-related risk factors, such as high blood pressure, smoking, obesity [24], and psychosocial stressors, such as racism [25].

### Native Hawaiians and Racism

To date, four studies have examined the effects of perceived racism on the health and well-being of Native Hawaiians. These studies found that levels of perceived racism were significantly associated with self-reported hypertension, higher systolic blood pressure (SBP), lower diurnal cortisol levels, and body mass index (BMI) [26–28]. In addition, Native Hawaiians who strongly identified with being Native Hawaiian perceived more racism than those who did not. There has only been one study that examined the relationship between perceived discrimination and mental health in Native Hawaiians. This study found a modest but significant correlation (*r* = 0.32) between perceived everyday discrimination and depressive symptoms. The most commonly reported reasons for participants’ experiences of discrimination were race, ancestry, or national origins followed by education and income levels [29].

### Cardiovascular Responses to Stressors

Numerous studies have found that exaggerated cardiovascular responses to, or impaired recovery following stressful events, are risk factors for CVD and hypertension [30–32] and can predict future CVD morbidity and mortality [33]. To date, few studies have examined the relationship between perceived racism and cardiovascular reactivity and recovery, with most primarily focused on African Americans (e.g., [34–43]). These studies suggest that the degree to which individuals perceive racism is significantly correlated with BP responses to lab stressors, allostatic load [1], and baseline levels of other psychophysiological measures (e.g., heart rate (HR) variability, galvanic skin response) over a prolonged period [25]. Allostatic load is a conceptual framework that describes the cumulative strain on the body when it is forced to adapt to adverse and chronic psychosocial and physical stressors [44]. When the body can no longer efficiently turn on or shut down its physiological responses over a prolonged period, the results can be damaging and potentially lethal [44].

The most common psychophysiological measures of the effects of exposure to stressors are peak cardiovascular reactivity and recovery rates [45]. Many studies have suggested that elevated and prolonged cardiovascular reactivity to an acute stressor is a marker for elevated hypertension, atherosclerosis, cardiac hypertrophy, and CVD mortality [32]. The strength and direction of the relationships between cardiovascular reactivity to a laboratory stressor and perceived racism have varied across studies. Diastolic blood pressure (DBP) reactivity appears to be most strongly associated with participants’ degree of self-reported, perceived racist experiences (e.g., [20, 36]), followed by SBP and HR reactivity (e.g., [35, 40]).

Some researchers have suggested that post-stress recovery may be more important than cardiovascular reactivity as an indicator of CVD risk [40]. Prolonged recovery periods are considered markers of chronic sympathetic activation that cause down-regulation of beta-adrenergic receptors in the heart and peripheral vasculature [46]. This down-regulation may lead to reduced cardiac output and increased peripheral vascular resistance, which, if repeated or extended presentations of a stressor occurs, results in elevated BP levels over time [44, 46]. Other studies have suggested that post-stress time to recovery is associated with participants’ hypertension status [46]; with family history of CVD after controlling for baseline cardiovascular activity, body mass index (BMI), waist-to-hip ratio, and smoking status [47]; and with higher levels and increased progression of carotid atherosclerosis [48]. Overall, few studies have examined the relative importance of cardiovascular recovery and reactivity as determinants of hypertension risk or CVD development (e.g., [37, 40]).

To understand better how perceptions of racism may be affecting Native Hawaiians on a psychophysiological level, the goals of this study were to:

1. Determine if there are significant differences in the degree of cardiovascular reactivity to either a blatant or subtle racial stressor between Native Hawaiian college students who report high vs. low levels of perceived racist experiences
2. Determine if there are significant differences in the degree of cardiovascular recovery following exposure to a blatant or subtle racial stressor between Native Hawaiian college students who report high vs. low levels of perceived racist experiences
3. Determine if there are significant differences in the degree of subjective ratings of distress following exposure to a blatant or subtle racial stressor between Native Hawaiian college students who report high vs. low levels of perceived racist experiences
4. Examine the bivariate correlations among dependent (i.e., cardiovascular reactivity and recovery, subjective ratings of distress) and independent variables (i.e., levels of perceived racism)

## Methods

**Fig. 1:**
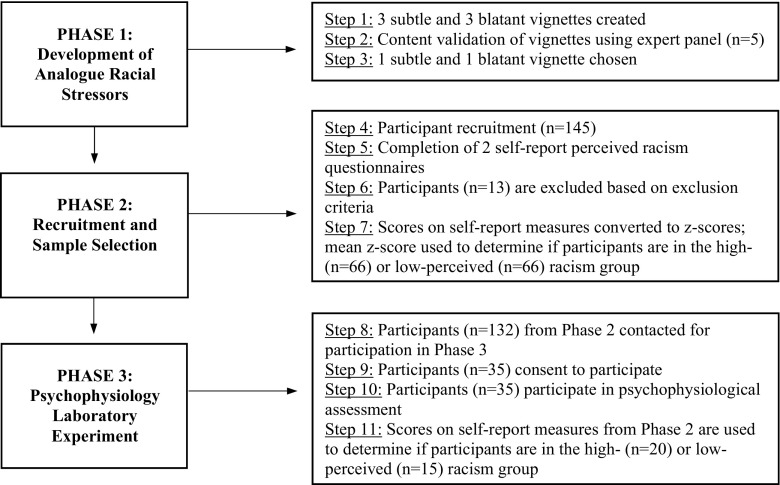
Overview of Methodology across Three-Phase Project

The study involved three sequential phases: (1) Development of Analogue Racial Stressors, (2) Recruitment and Sample Selection, and (3) Psychophysiology Laboratory Experiment. University of Hawai‘i Institutional Review Board approval was obtained for all three phases of this study. Figure 1 provides an overview of the methodology used across the three phases.

### Phase 1: Development of Analogue Racial Stressors

A total of six written vignettes that described racist behaviors toward Native Hawaiians were developed. The vignettes were based on an integration of current race-related political events, racist public statements, racist interpersonal experiences of Native Hawaiians, and examples of common racist experiences from the literature. Three different scenarios were used. One scenario described a negative interaction between a college student majoring in Education and the student’s college counselor. Another scenario described a negative interaction between a hotel manager and a guest. The third scenario involved a negative interaction between two individuals at a bar.

Two vignettes were written for each scenario: One vignette portrayed subtle racism, and one vignette portrayed blatant racism. Subtle racism is less obvious and involves omissions, inactions, or failure to help the targeted individual [49]. A subtle racist incident might involve a store clerk who follows a Native Hawaiian individual as he walks through the store but does not indicate explicitly why he does this. The targeted individual might assume he is being followed because he is Native Hawaiian. Blatant racism involves an overt intent to psychologically or physically harm the identified individual and is obvious to the person or group of people being discriminated against [49]. A blatant racist incident might involve a store clerk who says to the targeted individual, “Get out of here, Hawaiian. People like you are not welcomed in my store.” This individual would know he is being discriminated against because he is Native Hawaiian.

#### Content Validation of Vignettes

To examine the content validity of the subtle and blatant vignettes, snowball sampling was used to identify a panel of five experts in Native Hawaiian history, culture, or language or who have extensive experience working with Native Hawaiian individuals or communities. The panelists were asked to review and narrow down the original six vignettes to one blatant racist and one subtle racist vignette. An additional expert in measurement and psychophysiology research reviewed selected vignettes and provided feedback to ensure that the vignettes did not prompt a suggested emotional (e.g., “You are so angry at her.”) or physiological reaction (e.g., “Your heart is racing,” or “Your hands are clenched.”). Both vignettes were audiotape recorded by another individual who used a neutral tone and slow to normal speech rate. This audiotape recording was also reviewed by the expert in measurement and psychophysiology research to ensure the tone and speech did not prompt a suggested emotional response (e.g., [50, 51]).

### Phase 2: Recruitment and Group Assignment Based on Perceived Racism Scores

#### Participants

Native Hawaiian participants were recruited from the student population at a public university in Hawai‘i. A Native Hawaiian was defined as an individual who is a “descendant of the aboriginal people who resided in the islands now called Hawai‘i prior to 1778” (Title 45 CFR 1336.62 Subpart F). A convenience sample of 145 participants completed a brief screening questionnaire, a demographic questionnaire, the *Perceived Ethnic Discrimination Questionnaire-Community Version* (*PEDQ-CV*) [52], and the *Modified Oppression Questionnaire (OQ-MV)* [53]. Participants were provided with a gift card as a thank you for their participation in the study. Thirteen students were excluded for various reasons, such as not being Native Hawaiian (23.1%), endorsing Native Hawaiian ancestry but not identifying with being Native Hawaiian (30.8%), or not endorsing Native Hawaiian ancestry but identifying with being Native Hawaiian (15.4%). Thus, their data were not included in the sample. One hundred thirty-two participants met the eligibility requirements, which included having Native Hawaiian ancestry, identifying with being Native Hawaiian, being 18 years or older, not taking antihypertensive medications, and not ever being diagnosed with a serious medical condition (e.g., cardiovascular disease, diabetes, and severe mental illnesses). Persons using hypertension medications and those with a serious medical condition were excluded because their conditions could affect the results of the psychophysiological evaluation.

#### Self-Report Questionnaires and Measures

##### Demographic Questionnaire

The demographic questionnaire included 18 questions about the participant’s characteristics, such as sex, age range, BMI, ethnic background, primary ethnic identification, and self-reported family history of hypertension.

##### Perceived Racism Questionnaires

The *PEDQ-CV* [52] Lifetime Exposure Scale and the *OQ-MV* [53] were used to measure participants’ levels of perceived racism. The *PEDQ-CV* [52] is a 34-item self-report questionnaire, which has been used to measure perceived racism or ethnic discrimination across various ethnic groups. Each item is rated on a five-point Likert-type scale ranging from 1 (never happened) to 5 (happened very often) [52]. Total scores range from 34 to 170, with higher scores indicating higher levels of perceived ethnic discrimination. The Lifetime Exposure Scale has been shown to have good internal consistency (Cronbach’s alpha coefficients ≥ 0.95) with Black, Latino, and Asian American individuals [54]. The *PEDQ-CV* also demonstrated adequate convergent validity with the Symptom Checklist 90-Revised (SCL-90-R) depression scale (*r* = 0.38 to *r* = 0.47, *p* < 0.01) and the SCL-90 anxiety scale (*r* = 0.32 to *r* = 0.41, *p* < 0.01) for Chinese, Indian, and Filipino individuals [54].

The *OQ-MV* is based on the *Oppression Questionnaire* (OQ) [53]. Each item is rated on a four-point Likert-type scale ranging from 1 (not at all) to 4 (a great deal). This 11-item questionnaire was used in two previous studies that examined the impact of perceived racism on hypertension status [26] and physiological indices of stress [27] in community-based samples of Native Hawaiians. Total scores range from 11 to 44, with higher scores indicating higher levels of perceived oppression. In a previous study [26], the Cronbach’s alpha of this assessment instrument was 0.93.

##### Group Assignment of Participants Based on Baseline Levels of Perceived Racism

In order to minimize possible bias by the research assistants and participants, the participants’ (*n* = 132) assignment into either the high- or the low-perceived racism group based on their scores on the *PEDQ-CV* and *OQ-MV* were not shared until after their participation in the next phase of this study. The participants’ scores on the *PEDQ-CV* ranged from 33 to 131 with a mean score of 55.34 (standard deviation (SD) = 21.30). Scores on the *OQ-MV* ranged from 10 to 44 with a mean score of 22.84 (SD = 8.96). A Pearson’s correlation coefficient *r* was calculated for the *PEDQ-CV* and *OQ-MV* to determine the strength of the relationship between these two measures. The obtained correlation (*r* = 0.58, *p* < 0.001) indicated that these instruments measured similar but not identical constructs. The total scores were converted to their corresponding z-scores [55]. The mean of the two z-scores served as each participant’s final score and ranged from − 1.1855 to 2.9577. The median, *z* = − 0.158975, of the 132 participants was then used to assign the participants into two equal groups (*n* = 66 for each group). Individuals with scores higher than − 0.158975 were identified as persons with high levels of general perceived racism, while individuals with scores lower than − 0.158975 were identified as persons with general low levels of perceived racism.

### Phase 3: Psychophysiology Laboratory Experiment

#### Participants

One hundred and thirty-two Native Hawaiian university students from phase 2 were eligible and asked to participate in the experimental phase. Of the 132 participants asked, a total of 35 individuals agreed to participate. Fifteen (10 females; 5 males; all between the ages of 18 and 30) of the participants were identified to have low levels of perceived racism. Twenty (17 females, 3 males; 19 were between the ages of 18 and 30) of the participants who were identified to have high levels of perceived racism. These levels were based on data from the original sample in phase 2. Participants with low levels of perceived racism had a mean score of 19.07 (SD = 6.37, range 11–27) on the *OQ-MV* and a mean score of 39.60 (SD = 4.67, range 34–48) on the *PEDQ-CV*. Participants with high levels of perceived racism had a mean score of 30.55 (SD = 6.47, range 20–44) on the *OQ-MV* and a mean score of 64.10 (SD = 16.42, range 40–109) on the *PEDQ-CV*. Only 66% of participants who were identified to have low levels of perceived racism indicated Native Hawaiian as their primary ethnic identification versus 90% of participants who were identified to have high levels of perceived racism. All participants but one indicated that they were not current smokers. There were no significant differences in BMI of individuals in the low- and high-perceived racism groups. When asked about self-reported family history of hypertension, 27% of individuals identified to have low levels of perceived racism and 70% of individuals identified to have high levels of perceived racism said “yes.”

#### Psychophysiological Assessment

##### Cardiovascular Assessment

Cardiovascular measures of HR, SBP, and DBP were collected every 90 s throughout the experimental protocol using the Meditech ABPM-04 ambulatory blood pressure monitor (Meditech, Ltd.). HR, SBP, and DBP are defined as follows: HR is the number of heart beats per minute. SBP is the top number in a blood pressure reading and represents the pressure when the heart is working to pump blood to the rest of the body. DBP is the bottom number in a blood pressure reading and represents the pressure when the heart is resting and filling with blood [56].

##### Procedures

Prior to the lab visit, participants were asked to refrain from using alcohol, caffeine, and nicotine for at least 2 h before coming to the lab because of their potential effect on BP and HR. Participants were invited into the laboratory setting by two research assistants who were previously trained in the study protocol. The laboratory setting was a quiet, air-conditioned, medium-sized, office with a desk, three chairs, computer, and ambulatory blood pressure machine. Distractions were minimized. Participants were asked to sit on a comfortable chair while one of the research assistants reviewed the consent form and answered any questions of the participants.

After each participant provided informed consent, he or she was asked to sit with his or her feet flat on the floor and place his or her non-dominant arm on the desk next to him or her at the level of the right atrium. An appropriately sized occluding BP cuff that was connected to the Meditech ABPM-05 was placed on each participant’s non-dominant arm.

Each participant participated in a 6-min baseline period followed by two stimulus tasks; that is, task 1 = 3.5-min blatantly or subtly racist vignette + 60-s to respond to two questions about perceived racism as the motivating factor of the negative interaction and the degree of distress experienced, task 2 = 3.5-min blatantly racist or subtly racist vignette + 60-s to respond to two questions about perceived racism as the motivating factor of the negative interaction and the degree of distress experienced) interspersed with two 9-min resting periods [37]. Each participant was randomly assigned to one of two possible task orders (i.e., blatant racist vignette followed by subtle racist vignette or subtle racist vignette followed by blatant racist vignette) prior to the experimental protocol. The experimental protocol took a total of approximately 45 to 60 min to complete. Continuous HR and BP data were collected every 90 s during the following phases: baseline (6 min), task 1 (4.5 min), recovery 1 (9 min), task 2 (4.5 min), and recovery 2 (9 min).

##### Baseline

Each participant sat quietly during a 6-min baseline period without any distractions or tasks to collect baseline BP and HR data. During this time, the research assistants moved out of the view of the participant.

##### Task 1: Stressor Exposure

For task 1, the participant read a typewritten version of a 3.5-min blatant or subtle racist vignette while listening to an audiotape recording of the same vignette that was played using an audiotape recorder with a speaker. Immediately after simultaneously reading and listening to the vignette, participants read a question that was also read aloud by a research assistant. The first question was: “To what degree do you perceive racism to be the motivating factor in the treatment of the Native Hawaiian individual in the vignette presented?” Possible responses were indicated on a four-point scale from 1 (not at all) to 4 (an extreme amount), and participants circled the appropriate number. Immediately after the participants circled their response, they read another question that was also read aloud by a research assistant. The second question was “To what degree were you distressed by this scenario?” Possible responses were indicated on a four-point scale from 1 (not at all) to 4 (an extreme amount). Their BP and HR were measured every 90 s.

##### Recovery 1: Post-Stressor Termination

Each participant sat quietly for 9-min without any distractions or tasks to collect post-stressor recovery BP and HR data. During this time, the research assistants moved out of the view of the participant.

##### Task 2: Stressor Exposure

For task 2, the participants read a typewritten version of the 3.5-min, blatant or subtle vignette that was not presented during task 1 while listening to an audiotape recording of the same vignette. The same questions that were included in task 1 were read by participants as they were read aloud by a research assistant. Participants circled the appropriate numbers corresponding to their responses.

##### Recovery 2: Post-Stressor Termination

Each participant sat quietly for 9-min without any distractions or tasks to collect post-stressor recovery BP and HR data. During this time, the research assistants moved out of the view of the participant.

### Data Reduction and Analytic Strategy

*Baseline SBP*, *DBP*, and *HR* readings were computed by taking the mean of the readings during the last 3 min of the baseline period [40].

*Cardiovascular reactivity* scores for the first stressor task were calculated by subtracting the mean cardiovascular measure (i.e., SBP, DBP, or HR) during the baseline from the peak cardiovascular measure during the first stressor task (e.g., highest DBP reading during task 1—mean DBP during the baseline; e.g., [57]). Cardiovascular reactivity scores for the second stressor task were calculated by subtracting the mean cardiovascular measure of the first recovery period from the peak cardiovascular measure of the second stressor task (e.g., highest HR reading during task 2—mean HR during recovery 1). Peak SBP, DBP, and HR were defined as the highest SBP, DBP, or HR reading during each of the two stressor tasks.

*Cardiovascular recovery* scores were calculated by subtracting the mean cardiovascular measure during the recovery periods following each stressor task from the peak cardiovascular measure (i.e., SBP, DBP, or HR) during each stressor task.

### Statistical Analysis

All analyses were conducted using SPSS Version 19.0. A 2 (group) × 5 (condition) mixed, between-within subjects analysis of variance (ANOVA) was conducted for SBP, DBP, and HR. A 2 (group) × 2 (stressor type) ANOVA was conducted for subjective distress. The main focus of this study was to determine if there were significant main effects for “group,” which would indicate differences in physiological or subjective responses to vignettes between the high- and low-perceived racism groups across conditions. If there were main effects for groups or conditions, all possible pairwise comparisons were conducted post hoc.

Significant interaction effects between level (between group factor: high versus low levels of previous racist experiences) of perceived racism and condition (within-group factors of experimental condition: baseline, task 1, recovery 1, task 2, recovery 2) would indicate significant differences across conditions as a function of group membership. If an interaction was significant, independent samples *t* tests were conducted post hoc. Significant interaction effects between level (between group factor: high versus low levels of previous racist experiences) of perceived racism and stressor type (within-group factor: blatant versus subtle racial stressor) for subjective distress would indicate significant differences in subjective distress related to stressor type as a function of group membership. ANOVA, rather than MANOVA, were used because HR, BP, and measures of subjective distress are often not highly correlated [58]. Pearson’s correlations were conducted to examine the relationships between independent and dependent variables.

Missing cardiovascular measures were replaced with the median of two valid adjacent measures (i.e., one measure above and one below the missing value). There were 71 (3.07%) randomly distributed missing values out of 2310 total cardiovascular data points. Missing values were primarily due to a temporary malfunction of the cardiovascular monitoring equipment.

## Results

### Means and Standard Deviations of Dependent Variables

**Fig. 2:**
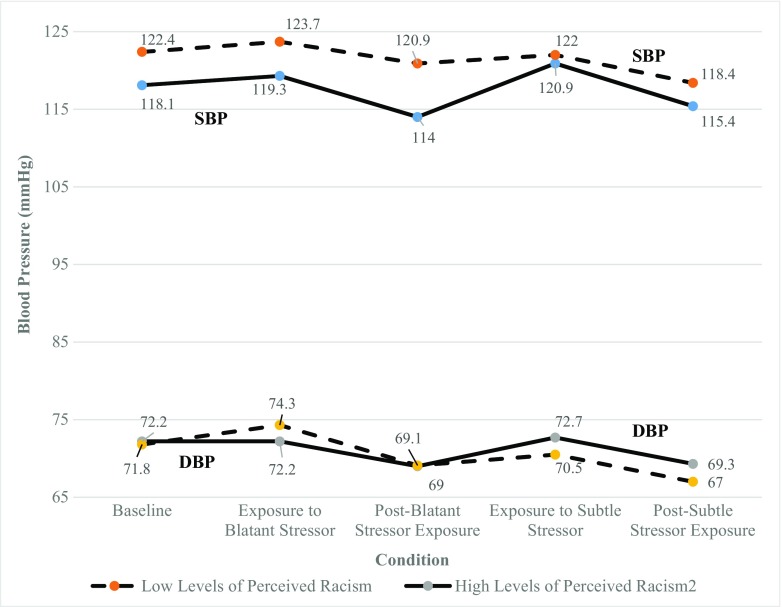
**Blood pressure (mmHg) readings across conditions by levels of perceived racism.** *mmHg* millimeters of mercury, *DBP* diastolic blood pressure; *SBP* systolic blood pressure; level of perceived racism based on average of z-scores on the Perceived Ethnic Discrimination Questionnaire-Community Version and Oppression Questionnaire-Modified Version

**Fig. 3:**
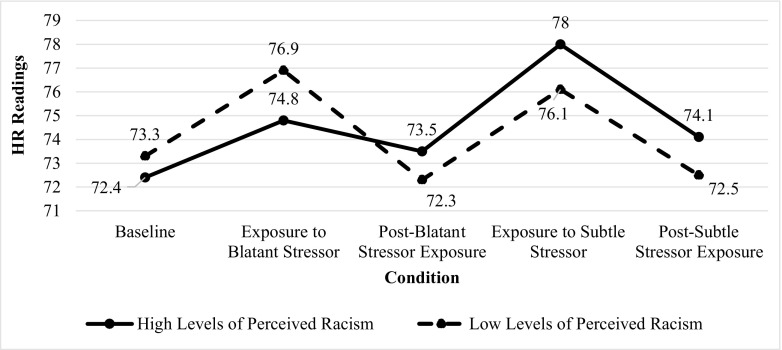
**Heart rate (bpm) readings across conditions by levels of perceived racism**. *bpm* beats per minute; level of perceived racism based on average of z-scores on the Perceived Ethnic Discrimination Questionnaire-Community Version and Oppression Questionnaire-Modified Version

The means and SDs for BP, HR, and subjective distress were calculated across each condition for all participants in each group (i.e., high or low baseline levels of perceived racism) and are presented in Table 1. Figures 2 and 3 illustrate the cardiovascular measures (BP and HR) across conditions for each group.

**Table 1:** Means and standard deviations of cardiovascular measures and subjective distress by levels of perceived racism and condition

Condition	Baseline perceived racism	Mean systolic blood pressure (mmHg) (SD)	Mean diastolic blood pressure (mmHg) (SD)	Mean heart rate (bpm) (SD)	Mean subjective distress (SD)
Baseline	Low	122.4 (14.4)	71.8 (8.0)	73.3 (10.5)	–
	High	118.1 (14.0)	72.2 (7.6)	72.4 (10.7)	–
	Total	120.0 (14.11)	72.0 (7.7)	72.8 (10.5)	–
Exposure to blatant stressor	Low	123.7 (16.3)	74.3 (8.2)	76.9 (12.4)	2.7 (0.7)
	High	119.3 (11.6)	72.2 (7.4)	74.8 (11.4)	3.1 (0.8)
	Total	121.2 (13.8)	73.1 (7.7)	75.7 (11.7)	2.9 (0.8)
Post-blatant stressor exposure	Low	120.9 (15.0)	69.1 (7.6)	72.3 (11.6)	–
	High	114.0 (11.3)	69.0 (8.2)	73.5 (10.9)	–
	Total	117.0 (13.3)	69.1 (7.8)	73.0 (11.1)	–
Exposure to subtle stressor	Low	122.0 (13.8)	70.5 (8.1)	76.1 (11.5)	2.2 (0.9)
	High	120.9 (12.3)	72.7 (7.4)	78.0 (12.4)	2.3 (0.9)
	Total	121.3 (12.8)	71.7 (7.7)	77.2 (11.9)	2.3 (0.9)
Post-subtle stressor exposure	Low	118.4 (10.8)	67.0 (8.1)	72.5 (11.2)	–
	High	115.4 (11.4)	69.3 (7.5)	74.1 (10.3)	–
	Total	116.7 (11.1)	68.3 (7.7)	73.4 (10.6)	–

Level of baseline perceived racism based on average of z-scores on PEDQ-CV and OQ-MV; subjective distress was measured by the query, “To what degree were you distressed by this scenario?” on a scale of 1 (Not at all) to 4 (An extreme amount) immediately after exposure to each racial stressor. Reactivity to Blatant/Subtle Stressor = Peak BP or HR reading during stressor-Mean BP or HR during baseline prior to stressor exposure. Recovery from Blatant Stressor = Mean BP or HR reading during the post-stress recovery period following stressor exposure-Peak BP or HR reading during stressor

*mmHg* millimeters of mercury, *bpm* beats per minute, *SD* standard deviation

### Systolic Blood Pressure Associated with Group and Condition

Figure 2 shows the means of SBP readings across conditions for all of the participants for the high- and low-perceived racism groups. There were significant effects for conditions (*F* = 9.76, *p* < 0.00) but non-significant effects for group (*F* = 0.86, *p* = 0.36) and group × conditions interactions (*F* = 2.31, *p* = 0.07). There were no significant differences in SBP between the high- and low-perceived racism groups. All pairwise comparisons were conducted post hoc using the Bonferroni correction with an adjusted criterion of *α* = 0.005 to determine significance. These post hoc comparisons were significant: (1) SBP recovery following blatant stressor exposure (mean difference (MD) = −4.2 mmHg; *p* = .000); (2) SBP recovery following subtle stressor exposure (MD = − 4.6 mmHg, *p* = 0.000); and (3) mean SBP following subtle stressor exposure and peak SBP during blatant stressor exposure (− 4.5 mmHg, *p* = 0.004).

### Diastolic Blood Pressure Associated with Group and Condition

Figure 2 shows the means of DBP readings across conditions for all of the participants for the high- and low-perceived racism groups. There were significant effects for conditions (*F* = 24.95, *p* < 0.000) and group × conditions interactions (*F* = 4.75, *p* < 0.001), but non-significant effects for group (*F* = 0.04, *p* = 0.84). Independent samples *t* tests were conducted post hoc to examine the degree to which DBP readings were affected by each condition as a function of group membership. These tests revealed no significant interactions when comparing DBP readings between the high- and low-perceived racism groups.

### Heart Rate Associated with Group and Condition

Figure 3 shows the means of HR readings across conditions for all of the participants for the high- and low-perceived racism groups. There were significant effects for conditions (*F* = 15.69, *p* < 0.001) and group × conditions interactions (*F* = 3.21, *p* < 0.02), but non-significant effects for group (*F* = 0.00, *p* = 0.93). Independent samples *t* tests were conducted post hoc to examine the degree to which HR readings were affected by each condition as a function of group membership. These tests revealed no significant interactions when comparing HR readings between the high- and low-perceived racism groups.

### Subjective Distress Associated with Group

A 2 (group) × 2 (stressor type) ANOVA was conducted to compare the mean ratings of subjective distress following stressor exposure for all participants for the high- and low-perceived racism groups. There were significant effects for stressor type (*F* = 17.38, *p* < 0.001), and non-significant effects for group (*F* = 1.00, *p* = 0.32) and group × stressor type (*F* = 0.70, *p* = 0.41).

### Degree to which Participants Perceived Racism to be the Motivating Factor

In order to examine participants’ perceptions about the racial stressors, the question “To what degree do you perceive racism to be the motivating factor in the treatment of the Native Hawaiian individual in this vignette?” was asked immediately after listening to and reading the blatant and subtle vignettes. Possible response options included “not at all,” “slightly,” “a moderate amount,” or “an extreme amount.” Thirty-three (94%) participants answered “an extreme amount” and two (6%) participants answered “a moderate amount” following exposure to the blatant stressor. No participants answered “not at all” or “slightly.” After exposure to the subtle stressor, the majority of participants (57%) answered “a moderate amount,” followed by “slightly” (31%) and “not at all” (6%) or “an extreme amount” (6%).

### Bivariate Correlations between Mean Baseline Perceived Racism Scores, SBP, DBP, and HR Reactivity and Recovery Scores and Subjective Ratings of Distress

Pearson’s *r* correlations were calculated to explore the bivariate relationships between mean baseline perceived racism scores and mean SBP, DBP, and HR reactivity to the blatant or subtle stressor exposure, mean SBP, DBP, and HR recovery from the blatant or subtle stressor, or subjective ratings of distress following exposure to either the subtle or blatant stressor (see Table 2). Notable significant correlations were found between baseline perceived racism levels and SBP reactivity to exposure to the subtle stressor (*r* = 0.36, *p* < 0.05) and DBP recovery following exposure to the blatant stressor (*r* = 0.37, *p* < 0.05) as well as subjective ratings of distress following exposure to the blatant stressor and subjective ratings of distress following exposure to the subtle stressor (*r* = 0.37, *p* < 0.05).

**Table 2:** Bivariate correlations between mean baseline perceived racism scores, systolic blood pressure (SBP), diastolic blood pressure (DBP), and heart rate (HR) reactivity and recovery scores and subjective ratings of distress

	Bl SBP R1	Bl SBP R2	Su SBP R1	Su SBP R2	Bl DBP R1	Bl DBP R2	Su DBP R1	Su DBP R2	Bl HR R1	Bl HR R2	Su HR R1	Su HR R2	Bl Sub Dis	Su Sub Dis
Perceived racism	− 0.11	− 0.18	0.36*	− 0.14	− 0.28	0.37*	0.09	0.08	0.02	0.16	0.20	− 0.13	0.23	0.06
Blatant (Bl) SBP reactivity		− 0.51**	− 0.18	− 0.03	− 0.06	− 0.28	0.30	− 0.14	0.10	0.16	− 0.04	− 0.19	0.24	0.20
Bl systolic blood pressure (SBP) recovery			− 0.44**	0.42*	0.16	0.16	− 0.49**	0.48**	0.18	− 0.34*	0.08	0.11	− 0.11	− 0.01
Subtle (Su) SBP reactivity (R1)				− 0.66**	− 0.14	0.12	0.45**	− 0.31	− 0.15	0.31	0.07	− 0.16	− 0.00	− 0.14
Su SBP recovery (R2)					0.08	0.29	− 0.45**	0.54**	0.08	− 0.08	− 0.06	0.20	− 0.17	0.02
Bl diastolic blood pressure (DBP) R1						− 0.35*	− 0.18	0.02	0.25	− 0.19	− 0.31	0.32	− 0.01	− 0.01
Bl DBP R2							− 0.38*	0.32	− 0.06	0.19	− 0.08	0.17	− 0.08	0.00
Su DBP R1								− 0.57**	− 0.10	0.32	0.13	− 0.31	0.13	0.11
Su DBP R2									0.05	− 0.19	0.27	− 0.05	− 0.04	− 0.12
Bl heart rate (HR) R1										− 0.72**	0.31	− 0.27	− 0.02	0.11
Bl HR R2											− 0.41**	0.30	0.17	0.08
Su HR R1												− 0.87**	− 0.16	0.07
Su HR R2													0.01	0.01
Bl subjective distress (SubDis)														0.37*

Perceived racism: Level of baseline perceived racism based on average of z-scores on PEDQ-CV and OQ-MV; Reactivity to Blatant/Subtle Stressor = Peak BP or HR reading during stressor-Mean BP or HR during baseline prior to stressor exposure. Recovery from Blatant Stressor = Mean BP or HR reading during the post-stress recovery period following stressor exposure-Peak BP or HR reading during stressor; SubDis was measured by question “To what degree were you distressed by this scenario

*mmHg* millimeters of mercury, *Bl* blatant, *Su* subtle, *SBP* systolic blood pressure, *DBP* diastolic blood pressure, *HR* heart rate, *R1* reactivity, *R2* recovery, *SubDis* subjective distress

*Correlation is significant at the 0.05 level (two-tailed). **Correlation is significant at the 0.01 level (two-tailed)

## Discussion

Our study is the first to explore the relationship between perceived racism and cardiovascular reactivity and recovery to racial scenarios with an indigenous population, Native Hawaiians, using an experimental design. There were two key findings of this study and a few interesting trends in the data worth mentioning. The first key finding is that SBP recovery following exposure to both the blatant and subtle racial stressors was significant, with a greater degree of recovery following subtle stressor exposure than that following blatant stressor exposure for high- and low-perceived racism groups. This finding is unexpected as individuals who report more experiences with racial discrimination tend to demonstrate a lesser degree of recovery following acute stressor exposure than those who report less experiences [23, 36–38, 40]. However, this finding may be a function of the age (34 of 35 participants were between the ages of 18 and 30) of participants in this study. Research indicates that SBP and DBP are independently associated with CVD risk for individuals, 50 years old or younger [59]. At the age of 50, elevated SBP is a more accurate predictor of CVD risk and mortality among individuals with hypertension than DBP because older individuals may experience stiffening in their arterial trees, whereas younger individuals tend to have highly distensible aortas [59]. Future studies should include a larger sample size with more variability in age to examine potential age effects.

The second key finding was that significantly greater subjective distress was reported following exposure to the blatant than to the subtle stressor for all participants. Overall, participants’ ratings of subjective distress were generally consistent with the pattern of reactivity observed in the low-perceived racism group, but less consistent with the pattern of reactivity observed in the high-perceived racism group. The discrepancy observed in the high-perceived racism group suggests that individuals with high levels of perceived racism may not be as aware of how much they are affected by subtle forms of racism. This is demonstrated by their cardiovascular responses to the subtle racial stressor. A lack of understanding about one’s own reactions to racial stressors may be a barrier to developing or utilizing effective coping strategies during stressful situations. Assisting participants with better understanding this relationship may potentially improve their ability to cope with racist stressors in the future.

Although not statistically significant, trends in the data suggested that the high-perceived racism group demonstrated greater reactivity to exposure to the subtle stressor than to the blatant stressor, incomplete HR recovery following exposure to both stressors, and incomplete SBP and DBP recovery following exposure to the subtle stressor. These trends may be consistent with findings from other studies, which indicate that individuals who have more experiences with perceived racism are more likely to have heightened sensitivity to unfair treatment [12] and interpret ambiguous, interpersonal interactions to be due to racism than individuals who have not reported previous experiences with racism [37]. Research also suggests that incomplete BP and HR recovery following stressor exposure is a stronger predictor of future BP levels than reactivity. Prolonged recovery, as indicated by these trends, suggests that individuals who take longer to recover from racist stressors may be more biologically reactive to these stressors than individuals who recover normally [60]. This corresponds with the allostatic load framework, which suggests that prolonged exposure to racial discrimination may interfere with the body’s ability to maintain homeostasis when stressors persist after the need for physiological activation has passed. Prolonged activation, which is also suggested in these data, implies the involvement of cognitive variables, such as perseveration or appraisal of control and ability to cope with the emotional reactions to a particular scenario [60]. If these findings were significant, they suggest that previous experiences with racial discrimination may place individuals at risk for earlier onset and increased severity of CVD later in life [61].

This study is not without limitations. One potential limitation is the generalizability of these study findings to real-life situations because of its experimental laboratory methodology. Despite the disadvantages (e.g., observation of only short-term stress responses, use of artificial stimuli that may not relate to real world experiences) of this methodology, previous studies have identified laboratory responses to stressors as valid markers for real-life responses [62]. A laboratory setting also provides the ability to implement strict environmental control [63], observe low frequency events and behaviors, and monitor changes in physiological responses [17] while monitoring or eliminating potential confounding factors.

Another limitation is the potential for reverse causality of increased stress reactivity. Specifically, individuals who are at risk for hypertension tend to show raised stress reactivity. Therefore, elevated stress reactivity may indicate a pre-existing risk for hypertension [64]. This study attempted to control for this possibility by using a convenience sample of normotensive, Native Hawaiian participants who were recruited from a large, public university on the island of Oʻahu. Although we controlled for this possibility, participants were primarily females (77.1%) who ranged in age from 18 to 30 years (97.1%). These demographics may limit the generalizability of these findings to males or younger and older Native Hawaiians.

Finally, the sample size was inadequate to examine the effects of commonly examined covariates (e.g., BMI, family history of hypertension, age, smoking status) and moderator and mediator variables (e.g., coping skills/style, social support) on the dependent variables. However, this is a preliminary study and the first of its kind to be conducted with a native population. Future studies should include a large enough sample size to determine whether similar patterns emerge and to control for key factors that have been found in previous studies conducted with other ethnic groups to impact the relationship between perceived racism and cardiovascular reactivity and recovery.

### Future Directions

The implications of our results can be used to address some of the CVD health disparities evident in this group and possibly prevent the future development of CVD for some Native Hawaiian individuals. Addressing the negative consequences of perceived racism on the cardiovascular health of Native Hawaiian individuals may require a multidisciplinary and multilevel approach targeted at at-risk individuals. This approach could include various components such as classroom-level or healthcare interventions, diversity training courses, media campaigns, and policy changes. In the classroom or a physician’s office, students and patients could be educated about common physiological responses to racial stressors and taught about effective ways of coping with them. Diversity training programs should also be required for all individuals who will work at an institution or company in Hawaiʻi. These programs could include anti-racism education and discussions around interventions to eliminate perceived racism toward Native Hawaiians in classrooms, institutions, and the larger community. Programs could emphasize the relationship between physiological responses to racial stressors and their potential negative health outcomes (e.g., CVD). Media campaigns could also be developed to educate the community about how perceived racism can physiologically affect one’s health and what one can do about it. Efforts to pass policies using results from these types of study could be used to emphasize the need to change the negative impact of various environmental and cultural factors (e.g., low paying jobs, low-income housing, restrictions placed on participation in cultural practices) disproportionately experienced among Native Hawaiian individuals [65]. Through these initiatives, we may be better able to increase awareness among Native Hawaiian individuals, and ultimately, regulate their physiological responses to all forms of racial stressor.
